# Catalytic Oxidation of Ammonia over Cerium-Modified Copper Aluminium Zinc Mixed Oxides

**DOI:** 10.3390/ma14216581

**Published:** 2021-11-03

**Authors:** Sylwia Górecka, Kateřina Pacultová, Dagmar Fridrichová, Kamil Górecki, Tereza Bílková, Radim Žebrák, Lucie Obalová

**Affiliations:** 1 Institute of Environmental Technology, CEET, VSB-Technical University of Ostrava, 17. Listopadu 15/2172, 708 00 Ostrava-Jih, Czech Republic; katerina.pacultova@vsb.cz (K.P.); dagmar.fridrichova@vsb.cz (D.F.); kamil.maciej.gorecki@vsb.cz (K.G.); tereza.bilkova@vsb.cz (T.B.); lucie.obalova@vsb.cz (L.O.); 2 Dekonta a.s., Dřetovice 109, 273 42 Stehelčeves, Czech Republic; radim.zebrak@dekonta.cz

**Keywords:** ammonia oxidation, mixed metal oxides, zinc, copper, copper-cerium catalysts, Cu-Zn, Cu-Ce

## Abstract

Copper-containing mixed metal oxides are one of the most promising catalysts of selective catalytic oxidation of ammonia. These materials are characterized by high catalytic efficiency; however, process selectivity to dinitrogen is still an open challenge. The set of Cu-Zn-Al-O and Ce/Cu-Zn-Al-O mixed metal oxides were tested as catalysts of selective catalytic oxidation of ammonia. At the low-temperature range, from 250 °C up to 350 °C, materials show high catalytic activity and relatively high selectivity to dinitrogen. Samples with the highest Cu loading 12 and 15 mol.% of total cation content were found to be the most active materials. Additional sample modification by wet impregnation of cerium (8 wt.%) improves catalytic efficiency, especially N_2_ selectivity. The comparison of catalytic tests with results of physicochemical characterization allows connecting the catalysts efficiency with the form and distribution of CuO on the samples’ surface. The bulk-like well-developed phases were associated with sample activity, while the dispersed CuO phases with dinitrogen selectivity. Material characterization included phase composition analysis (X-ray powder diffraction, UV-Vis diffuse reflectance spectroscopy), determination of textural properties (low-temperature N_2_ sorption, scanning electron microscopy) and sample reducibility analysis (H_2_ temperature-programmed reduction).

## 1. Introduction

Ammonia (NH_3_) is a colorless gas with a characteristic expressive odor. Its emissions are estimated at approximately 5600 kt y^−1^ [1]. NH_3_ contributes to the acidification of soil and water due to its deposition, eutrophication in aquatic ecosystems and the excess of nitrogen. It also participates in the formation of acid rains and photochemical smog or ozone depletion [2,3,4]. In the human body, it affects the respiratory tract, lung functions, causes eye and skin irritation, and at higher doses, can cause death [5,6].

The production of NH_3_ is mostly related to agriculture; in developed countries, 80–90% of ammonia emissions come from animal husbandry, fertilizer manufacturing, and biomass fuel combustion. Another source of ammonia emissions is related to the industry, including energy production, transport, industrial processes (chemical, pharmacy, plastics) or selective catalytic reduction (SCR) and selective non-catalytic reduction (SNCR) technology for NOx abatement [7,8].

One of the most promising methods of ammonia emissions limitation, apart from adsorption and NH_3_ catalytic decomposition [5,9,10], is selective catalytic oxidation of ammonia to dinitrogen and water vapor (NH_3_-SCO). The reaction proceeds according to Equation (1) [7].

4NH_3_ + 3O_2_ → 2N_2_ + 6H_2_O (1)

Many catalysts have already been tested for NH_3_-SCO [7], e.g., transition metal oxides [6,8,11,12,13], rare earth oxides [8,14], supported noble metals [15,16,17,18] and transition metals [19], metal modified oxides [20,21], zeolites [22,23,24], mesoporous silica [25] and clays [26]. Most of those catalysts showed very good activity; however, ensuring high N_2_ selectivity is still one of the main challenges. Side reactions may occur to produce NO and N_2_O as undesired products [5].

Among all catalysts listed above, Cu-based materials show good efficiency, and they are still intensively studied. Many types of Cu-containing catalysts have been tested in the last years, showing high activity and selectivity in ammonia oxidation reactions [6,8,19,24,27,28,29]. The catalytic efficiency of such materials is usually affected by many factors, such as Cu content [8,29,30,31], preparation method [32], calcination temperature [6,30,31], particle size [32] or type of support [33]. However, the copper oxide was often proved to be an active species in the NH_3_-SCO reaction [5,8,29,31,34]. Thus, the optimal amount of Cu, as well as nature/type of copper phase, dispersion level, reducibility, and a large number of accessible acidic surface sites, are the parameters that define its catalytic performance [5].

In our previous study [8,29], Cu-Mg-Al-(Ce) and Cu-Mg-Fe-(Ce) mixed oxides prepared by hydrotalcite-like precursors, were tested to obtain the NH_3_-SCO catalyst, which would be active and sufficiently selective at temperatures up to 350 °C. The best catalytic performance was achieved using catalysts calcined at 800 °C with Cu loading of 5 and 7 mol.% of total cation content. The catalytic activity and selectivity of Cu-containing mixed metal oxides were connected to the well-dispersed CuO phase for both materials series. The formation of the CuO phase was affected by the level of Cu loading. Higher Cu content, however, caused crystal growth connected with a specific surface area (*S*_BET_) decrease, which lead to the drop in N_2_ selectivity. With increasing Cu loading, the catalyst’s reducibility changed and the amount of reducible species increased. Simultaneously, for the Ce-doped Cu-Mg-Al samples, the synergic effect of Cu and Ce, due to the change in near-surface chemical states caused by the formation of redox couples Cu^+^ + Ce^4+^ → Cu^2+^ + Ce^3+^, was confirmed. The Ce addition affected the catalytic performance of the tested samples. It had a positive effect on NH_3_ conversion for samples with Cu loading of 5 and 7 mol.% of total cation content. A similar direct effect was not observed for the Ce-impregnated Cu-Mg-Fe mixed metal oxides.

Since the mixed metal oxides obtained by calcination of hydrotalcite-like compounds are very promising materials, they have gained the attention of many scientists. These kinds of materials are characterized by homogeneously dispersed active centers, thermal stability, and relatively high surface area [6,8,11,29,35,36]. There is still the effort to find an appropriate catalyst, which is sufficiently active and selective in NH_3_-SCO, by changing its chemical composition and, therefore, the physical-chemical properties.

The presented study is focused on the characterization, and catalytic efficiency of Cu-Zn-Al-O and Ce/Cu-Zn-Al-O mixed metal oxides (MMO), as catalysts of selective catalytic oxidation of ammonia to dinitrogen. Cu-Zn-Al MMO was chosen for this study for several reasons. According to the literature, the addition of Zn to the MMO structure increases the *S*_BET_ [37,38,39]. It also improves catalyst reducibility by decreasing the reduction temperature and increasing ZnO content, causing higher dispersion of the CuO phase [38,40] and reduction of the Cu particle size [38]. All these effects should be beneficial for NH_3_-SCO.

The tested materials were obtained by calcination of hydrotalcite-like materials at 800 °C to obtain a mixture of simple and complex oxide systems, like CuO, ZnO, ZnAl_2_O_4_-like spinel, and CeO_2_ in the case of the cerium-modified set of materials.

Our research shows that the proposed oxide systems exhibit very good catalytic efficiency at low temperatures for ammonia oxidation. The unmodified and Ce-modified samples present significantly different catalytic activity and selectivity that is strictly related to the samples’ phase composition. The best results were obtained for the Ce/Cu-Zn-Al-O materials. All samples reach 95–100% of ammonia conversion and exhibit N_2_ selectivity at approximately 35–40%.

## 2. Materials and Methods

### 2.1. Catalysts Preparation

The mixed metal oxides were obtained by the calcination of hydrotalcite-like compounds at 800 °C for 9 h in air. Part of the calcined material was additionally impregnated by Ce (Ce(NO_3_)_3_·6H_2_O, Penta). Precursors were synthesized via the co-precipitation method from aqueous solutions of Cu(NO_3_)_2_·6H_2_O (Penta), Zn(NO_3_)_2_·6H_2_O (Penta), Al(NO_3_)_3_·6H_2_O (Penta). The precursor synthesis procedure and catalyst preparation are described in [8]. Sample codes and intended compositions are shown in Table 1.

### 2.2. Sample Characterization

The chemical composition of hydrotalcite-like materials was determined by atomic absorption spectroscopy (AAS) with the use of an AnalyticJena CONTR AA700 spectrometer.

Microscopic investigations of the mixed metal oxides (chemical composition and surface imaging) were determined by means of scanning electron microscopy (SEM: Tescan Vega) with a tungsten cathode and energy dispersive X-ray spectroscopy (EDS: EDAX). The micrographs of the selected samples were obtained using secondary electron (SE) and backscattered electron (BSE) modes, with an acceleration voltage of 30 keV. Prior to analysis, the samples were gold-sputtered to ensure adequate electron conductivity.

The phase composition of the materials was determined by X-ray powder diffraction with the use of a Rigaku Smart Lab diffractometer.

The specific surface area (S_BET_) of the calcined samples was determined by the BET method using a 3Flex (Micromeritics) automated gas adsorption system.

The UV-Vis diffuse reflectance spectra of the calcined samples were recorded using a Shimadzu UV-2600 (IRS-2600Plus) spectrophotometer. The optical direct band gap was estimated based on Tauc’s plot.

The reducibility of mixed metal oxides was studied by temperature-programmed reduction (H_2_-TPR) using an AutoChem II 2920 (Micromeritics).

Further information on the measurement condition can be found in [8].

### 2.3. Catalytic Studies

The catalytic tests, selective ammonia oxidation, were performed in a quartz microreactor system under atmospheric pressure at temperatures from 250 °C up to 350 °C. The measurement conditions, sample preparation, and product detection has been reported in [8].

Ammonia conversion was calculated with the use of Equation (2):

(2) $${\left(NH_{3}\right)}_{conv}=\left(\frac{{\left[NH_{3}\right]}_{in}-{\left[NH_{3}\right]}_{out}}{{\left[NH_{3}\right]}_{in}}\right)\cdot 100\left(\%\right)$$

Process selectivity to the dinitrogen was calculated with the use of Equation (3):

(3) $${\left(N_{2}\right)}_{sel}=\left(1-\frac{{\left[NO\right]}_{out}+{\left[NO_{2}\right]}_{out}+2{\left[N_{2}O\right]}_{out}}{{\left[NH_{3}\right]}_{in}-{\left[NH_{3}\right]}_{out}}\right)\cdot 100\left(\%\right)$$

Process selectivity to side products was calculated with the use of Equation (4):

(4) $$Sel_{i}=\frac{{\left[C_{i}\right]}_{out}}{\sum {\left[C_{i}\right]}_{out}}$$

## 3. Results

### 3.1. Mixed Metal Oxides Characterisation

The 800-Cux-Zn-Al mixed metal oxides were obtained by calcination of hydrotalcite-like precursors at 800 °C. The basic characterization of layered materials is presented in the Supplementary Information (Table S1 and Figure S1). The 800-Ce/Cux-Zn-Al mixed metal oxides were obtained through impregnation of 800-Cux-Zn-Al samples by cerium and additional calcination at 800 °C. The total molar ratio of divalent (sum of Cu and Zn) and trivalent (Al) cations of unmodified samples is constant: 2.03. With increasing Cu mol.% loading, the Zn cations are substituted by Cu, while the Al remains constant. Sample codes and intended chemical compositions are summarized in Table 1.

#### 3.1.1. Chemical Composition

The chemical composition of the obtained metal oxides was described by atomic absorption spectroscopy (AAS) and energy-dispersive X-ray spectroscopy (EDS). The standard mineralization procedure, with the use of 36% solution of HCl, does not allow for total samples dissolving. Additional EDS measurements and X-ray diffraction analysis of the obtained precipitate—only undissolved phases (Supplementary, Table S2, Figure S2) reveal the chemical composition of divalent and trivalent cations close to those characterized by AB_2_O_4_ spinel forms, A/B = 0.5 (A—divalent, B—trivalent cations). Therefore, the precise measurement of chemical composition with AAS was impossible. Instead, the less precise surface analysis of sample composition via EDS was performed (Table 2). Due to EDS method limitations (local and surface analysis), the observed values could be affected by the elements’ exposure at the material’s surface, as well as the standard error of EDS measurements.

The comparison of intended Cu/Zn and Cu/Al molar ratios with calculated ones reveals discordance between Cu/Al int. and Cu/Al cal. values, while Cu/Zn, both, int., and cal., show quite a good accordance in the whole Cu amount range. It suggests that the distribution of zinc cations at the material’s surface is consistent with the assumptions, while the aluminum-enriched were at the samples’ surface. Surface enrichment by aluminum was also reported by Obalová et al. [41].

#### 3.1.2. Phase Composition and Morphology of Mixed Metal Oxides

**Figure 1:**
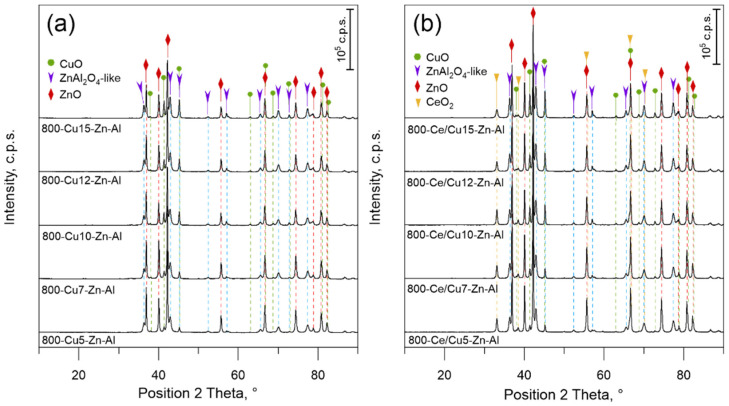
Phase composition of (**a**) 800-Cux-Zn-Al and (**b**) 800-Ce/Cux-Zn-Al mixed metal oxides.

The X-ray diffraction patterns (XRD) of 800-Cux-Zn-Al and 800-Ce/Cux-Zn-Al oxides are presented in Figure 1. Due to the high calcination temperature, the occurrence of patterns characteristic for simple and complex oxide systems is expected [8,42]. Indeed, the XRD analysis revealed the occurrence of ZnO, CuO, ZnAl_2_O_4_-like spinels and additionally CeO_2_ in the case of the 800-Ce/Cux-Zn-Al series. All samples, Ce-modified and un-modified, present a similar phase composition regardless the Cu mol.% loading. The diffraction lines occurring at approximately 37, 40, 42, 55, 66.5, 74, 79 and 82° 2θ correspond to zinc oxide (ICDD PDF-2 card No 01-080-0075), while lines at approximately 38, 41, 45, 63, 68.5, 73, 79 and 80° 2θ correspond to tenorite—CuO (ICDD PDF-2 card No. 01-080-1916). The estimated crystallite size of ZnO is about 32–45 nm and 38–50 nm for 800-Cux-Zn-Al and 800-Ce/Cux-Zn-Al respectively, while the crystallite size of CuO is approximately 26–41 nm and 30–41 nm for unmodified and Ce-modified samples, respectively. The intensity ratio of CuO and ZnO characteristic peaks (Table 3) grows with increasing Cu mol.% loading. It suggests that with increasing Cu mol % loading, the well-developed CuO particles are formed, influencing the intensity of characteristic CuO crystallographic pattern lines. Copper oxide crystallite size analysis (Table 3) shows that there is no visible trend related to Cu amount and size of CuO crystallites; however, it could be seen that the estimated size of CuO is the lowest for samples 800-Cu5-Zn-Al and 800-Ce/Cu5-Zn-Al and increased for samples with higher Cu content.

The diffraction lines characteristic for the gahnite-like phase (ZnAl_2_O_4_) were observed at approximately 36, 43, 52, 57, 65, 70 and 77.5° 2θ (ICDD Pdf-2 card No. 01-070-81-81). The estimated crystalline size of ZnAl_2_O_4_ is approximately 15–23 nm and 22–27 nm for 800-Cux-Zn-Al and 800-Ce/Cux-Zn-Al, respectively. Analysis of the gahnite and zinc oxide intensity ratio (*I*_G_) shows that the *I*_G_ values increase with increasing Cu mol.% loading (Table 3). The increase in ZnAl_2_O_4_ pattern line intensity is strictly connected with the improvement of gahnite phase crystallinity. The differentiation of characteristic lines of gahnite and CuAl_2_O_4_ or (Cu,Zn)Al_2_O_4_ is impossible from XRD measurements due to similar 2 Theta positions of the characteristic lines. However, it should be noted that part of the zinc cations could be substituted by copper, resulting in the formation of non-stoichiometric spinel-like phase.

Since copper and zinc are characterized by similar ionic radii in tetrahedral coordination, (0.057 nm and 0.060 nm, respectively) [43], none of the presented phases are stoichiometrically pure. Diffuse reflectance spectroscopy (UV-vis-DRS) and further analysis of optical characteristics with the use of Tauc’s plots allow us to estimate the direct optical band gaps (*E*_g_^d^) (Table 3). The obtained values correspond to the zinc oxide band gap *E*_g_^d^ = 3.26 eV [44]; however, slightly lowering the direct bandgap values with an increase in copper content in the case of sample set 800-Cux-Zn-A suggests a change of ZnO structure by partial substitution of Zn by Cu [44]. Due to the dominance of ZnO structure features in the UV-vis-DRS signal, the direct band gaps of CuO and ZnAl_2_O_4_-like spinel were not estimated. A similar substitution of Zn cations with Cu was observed for spinel-like structures. The EDS analysis of the precipitate obtained after material mineralization (Table S2), revealed that the AB_2_O_4_ spinel-like forms observed in the diffractogram are indeed composed of approximately 3–4 mol.% of Cu, where the sum of Cu, Zn and Al is 100 mol.%.

Our previous study, Górecka et al. [8], shows that the formation of CuO oxides after calcination of Cu-Mg-Fe hydrotalcite-like compounds is highly related to the copper concentration. Results show that in the case of samples with low copper loading below 7 mol.%, copper cations most likely form an amorphous structure or small, dispersed CuO particles that could not be detected by XRD methods. While current analysis reveals that even if part of zinc is substituted by copper in ZnO and/or ZnAl_2_O_4_-like forms, the rest of Cu most likely forms well-developed CuO oxide species regardless the copper amount. The CuO characteristic diffraction pattern lines are visible and intense even for samples with the lowest Cu loading. It suggests that the zinc–aluminum matrix favors the formation of CuO oxides instead of the incorporation of copper into other oxide species.

Additionally, diffraction patterns occurring at approximately 33, 38.5, 67 and 70° 2θ (Figure 1b) were ascribed to CeO_2_ (ICDD Pdf-2 card No. 01-078-5328). The crystallite size of cerium oxides was estimated at approximately 20–30 nm. The intensity ratio analysis of cerium oxide and zinc oxide shows quite constant values at about 0.10. However, it should be noted that in the case of sample 800-Ce/Cu10-Zn-Al, the calculated *I*_C_/*I*_Z_ ratio differs significantly from other values. This could be related to the distribution of CeO_2_ particles on the material’s surface.

**Figure 2:**
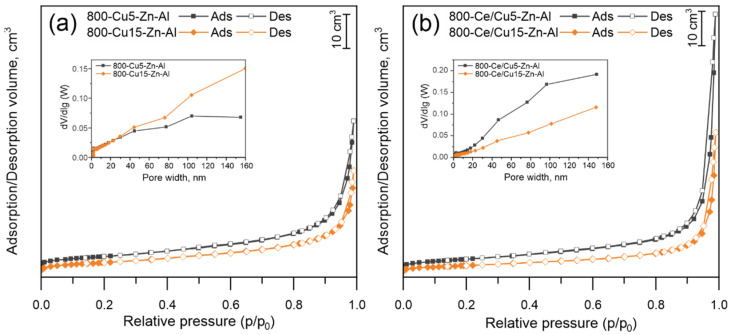
Textural properties of (**a**) 800-Cu5-Zn-Al and Cu15-Zn-Al, (**b**) 800-Ce/Cu5-Zn-Al and 800-Ce/Cu15-Zn-Al; Ads—adsorption curve, Des—desorption curve.

The low-temperature N_2_ adsorption BET analyses were performed to study the textural properties of 800-Cux-Zn-Al and 800-Ce/Cux-Zn-Al mixed metal oxides. The nitrogen adsorption/desorption isotherms of selected samples are presented in Figure 2, while the measured values of surface area and t-plot pore volume are presented in Table 3. The specific surface area calculated by means of the BET method was found to be lower than 22 m^2^ g^−1^. There is no visible trend connecting the increase in Cu mol.% loading and surface area; however, it could be seen that samples modified by Ce show lower BET surface areas in comparison to non-modified ones with the exception of 800-Ce/Cu10-Zn-Al sample. This could be related to the samples’ recalcination and possible surface covering (pore blocking) by Ce.

The estimated values of t-plot external surface area did not show any visible trend and remained at approximately 2–6 m^2^ g^−1^. The adsorption/desorption isotherms presented in Figure 2 represent a type IV isotherm with a small H3 hysteresis loop observed in the *p*/*p*_0_ range of 0.8–1.0. It indicates that samples belong to the mesopore group with groove pores structures [45]. The inset figure in Figure 2 shows the pore size curve, determined from the adsorption branch of the isotherm. It shows no observable monomodal mesoporosity within the sample. The adsorption/desorption isotherms of other un-modified and modified samples are presented in Figure S3, Supplementary Information.

Comparing the BET specific surface area of 800-Cu10-Zn-Al and 800-Ce/Cu10-Zn-Al with the *I*_C_/*I*_Z_ XRD intensity of the Ce-modified sample shows that Ce influences the material’s textural properties. The sample before impregnation was characterized by one of the lowest BET specific surface areas (16 m^2^ g^−1^) and t-plot external areas (2 m^2^ g^−1^) among the un-modified materials. After impregnation, the specific surface area slightly increased to 19 m^2^ g^−1^. Simultaneously, the *I*_C_/*I*_Z_ XRD intensity of 800-Ce/Cu10-Zn-Al significantly increased in comparison to other modified materials. It suggests that, as a result of impregnation, cerium oxide covers more surface area in comparison to other samples in the set.

**Figure 3:**
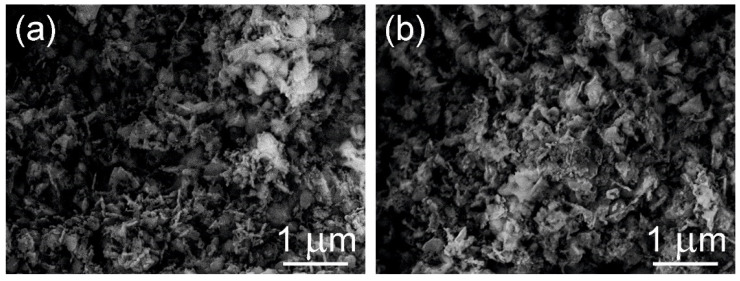
SEM micrographs of (**a**) 800-Cu10-Zn-Al and (**b**) 800-Ce/Cu10-Zn-Al, SE+BSE mode.

The micrographs of 800-Cu10-Zn-Al and 800-Ce/Cu10-Zn-Al (SEM, SE+BSE mode), presented in Figure 3 and Figure S4 (Supplementary Information), show the samples’ surface before and after impregnation. Both images present structures typical for mixed metal oxides; however, the 800-Ce/Cu10-Zn-Al sample represents a fluffier structure than others. The micrographs of other samples can be seen in Figure S5, Supplementary Information.

#### 3.1.3. Reducibility and Oxidation State of Cu- and Ce-Containing Oxides

**Figure 4:**
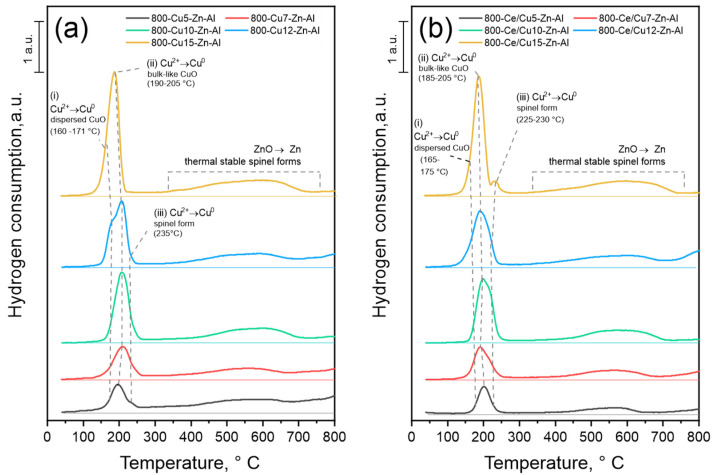
H_2_-TPR reduction profiles of (**a**) 800-Cux-Zn-Al and (**b**) 800-Ce/Cux-Zn-Al.

The reducibility behavior of 800-Cux-Zn-Al and 800-Ce/Cux-Zn-Al were investigated via the H_2_-TPR method. The reduction profiles, shown in Figure 4, present the influence of copper mol.% loading and cerium addition on the samples’ reducibility, while the theoretical and measured consumption of hydrogen are summarized in Table 4. Since the zinc–alumina matrix influenced the formation of CuO phases, the impact in material reducibility is expected.

The main reduction maxima was observed up to 250 °C and it is attributed to the reduction of Cu^2+^ to Cu^0^ in CuO and spinel-like compounds [8,46]. For both series of materials, the three reduction sub-maxima were found at approximately (i) 160–171, (ii) 195–205 and (iii) 230–235 °C for series 800-Cux-Zn-Al and at approximately (i) 165–175, (ii) 185–205 and (iii) 230 °C for series 800-Ce/Cux-Zn-Al. It could be seen that the position of reduction maxima in the case of Ce-modified samples is shifted to higher temperatures in comparison to the non-modified samples. It is probably related to the samples’ recalcination and the resulting increase in crystallinity [6]. Since the Cu^2+^ reduction in CuO and spinel-like phases takes place up to 250 °C [8,46], the occurrence of sub-maxima is related to different forms of both oxides and their reducibility [8]. Therefore, the first maximum (i) is attributed to the reduction of Cu^2+^ in highly dispersed CuO species, the second (ii) to copper reduction in bulk-like CuO, while the last maximum (iii) is related to the reduction of Cu^2+^ in spinel forms. The second maxima seem to become more visible and dominant with increasing Cu mol.% loading. The *I*_T_/*I*_Z_ ratio (see Table 3) shows that with increasing copper amount, the tenorite phase becomes more dominant in comparison to ZnO. The occurrence of bulk-like CuO forms was confirmed additionally by UV-Vis-DRS measurements (Figure S6, Supplementary Information). The intensity of the characteristic d-d transition of Cu^2+^ maxima at approximately 450–600 nm increases with the increase in Cu mol.% loading, suggesting the formation of Cu-O bulk-like particles for Cu-rich samples. Therefore, the increase in reduction maxima intensity around 185–200 °C remains in line with expectations. The broad reduction peak in the temperature range of 400–750 °C is related to the reduction of thermally stable spinel forms—(Cu, Zn)Al_2_O_4_, as well as the reduction of ZnO to metallic zinc [47]. The reduction maxima of CeO_2_ are not observable at the reduction profiles of 800-Ce/Cux-Zn-Al samples. It is possible that the characteristic CeO_2_ signal in the temperature range 400–500 °C [48] is covered by the signal registered for spinels and/or ZnO reduction. Comparison of the values summarized in Table 4 allows noticing that the measured hydrogen consumption (up to 300 °C) does not exceed 50% of theoretical H_2_ consumption, assuming that only Cu^2+^ cations occur and are reduced to Cu^0^. It means that copper can occur in different oxidation states other than Cu^2+^ and could form oxides like Cu_2_O, undetected by XRD measurements. It is also possible that part of the copper forms thermally stable spinel forms that are reduced above 300 °C.

### 3.2. Catalytic Tests

**Figure 5:**
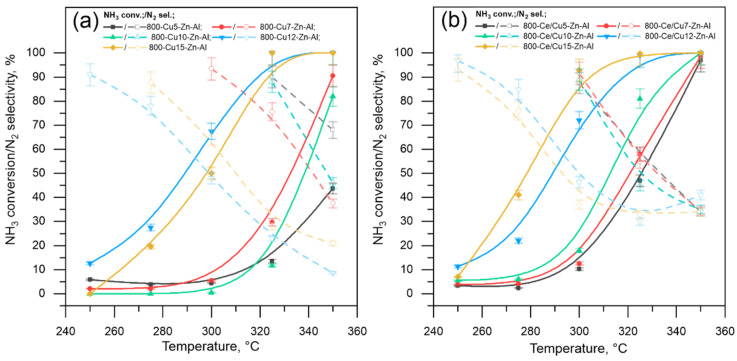
Catalytic test results for low catalytic oxidation of ammonia to dinitrogen performed over (**a**) 800-Cux-Zn-Al and (**b**) 800-Ce/Cux-Zn-Al mixed metal oxides; experimental conditions: catalyst mass = 200 mg, [NH_3_] = 0.035 mol.%, [O_2_] = 20 mol.%, [N_2_] = balance, total flow rate = 100 cm^3^ min^−1^, WHSV = 500 mL min^−1^ g^−1^.

The 800-Cux-Zn-Al and 800-Ce/Cux-Zn-Al mixed metal oxides were tested as catalysts for low-temperature ammonia oxidation—the experimental conditions, especially ammonia and oxygen mol.%, 0.035 and 20 mol.%, respectively, were chosen to simulate the oxygen reach flow. All tested materials showed relatively high catalytic activity in a temperature range up to 350 °C; however, process efficiency depends on copper mol.% loading and cerium addition (Figure 5).

Results of catalytic tests performed on unmodified samples (Figure 5a) show that the catalytic activity increases with the increase in copper mol.% loading, while the catalysts selectivity dropped. The presented results allow to divide the samples into two groups (i) 800-Cu5-Zn-Al, 800-Cu7-Zn-Al, 800-Cu10-Zn-Al and (ii) 800-Cu12-Zn-Al, 800-Cu15-Zn-Al. The first group (i) does not reach 100% of ammonia conversion up to 350 °C, with the best catalytic activity (approximately 90% of total ammonia conversion) observed for 800-Cu7-Zn-Al, while the sample with the lowest copper mol.% loading not reaching 50% of NH_3_ conversion at 350 °C. At the same time, dinitrogen selectivity drops from 70% to 35% for both 800-Cu5-Zn-Al and 800-Cu7-Zn-Al. A similar trend was observed for the second group of unmodified samples (ii). These materials allowed for a total ammonia conversion at 350 °C and generally exhibit higher activity within the whole temperature range compared to the other unmodified samples. Simultaneously, with increasing ammonia conversion, N_2_ selectivity drops to approximately 10 and 20% for 800-Cu12-Zn-Al and 800-Cu15-Zn-Al, respectively.

The results of catalytic performance over Ce-modified samples, presented in Figure 5b, show that wet impregnation of 800-Cux-Zn-Al materials by cerium improves catalytic efficiency of Cu-containing mixed metal oxides. Like in the case of unmodified samples, it is possible to divide the materials into two groups: (i) 800-Ce/Cu5-Zn-Al, 800-Ce/Cu7-Zn-Al, 800-Ce/Cu10-Zn-Al and (ii) 800-Ce/Cu12-Zn-Al, 800-Ce/Cu15-Zn-Al. All modified materials reach 100% of ammonia conversion at 350 °C; however, the first group of samples (i) shows lower catalytic activity in the whole temperature range in comparison to the second group of materials. Samples 800-Ce/Cu12-Zn-Al and 800-Ce/Cu15-Zn-Al show relatively high activity at lower temperatures, while in the case of samples with lower copper mol.% loading, the ammonia oxidation starts at approximately 300 °C showing NH_3_ conversion above 10%. Comparing the results from both modified and unmodified samples allowed noticing that the cerium addition improves catalyst activity within the whole temperature range. For all Ce-modified samples at 350 °C, the ammonia conversion level is higher than 95%. The most significant changes could be observed for 800-Ce/Cu5-Zn-Al. However, the increase in catalytic activity is accompanied by a decrease in selectivity to dinitrogen that at 350 °C is approximately 35%, i.e., almost 35% points lower in comparison to 800-Cu5-Zn-Al. In the case of the second group (ii) of Ce-modified materials, the addition of cerium significantly improves the materials’ selectivity. At 350 °C, the level of N_2_ selectivity is almost 10–20% pt. higher in comparison to unmodified catalysts.

**Figure 6:**
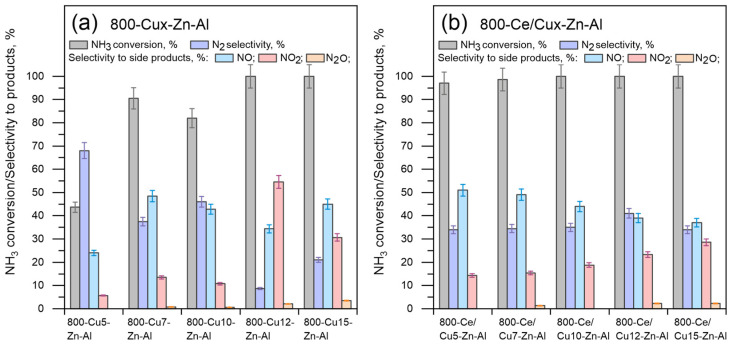
Catalytic test results for low catalytic oxidation of ammonia to dinitrogen performed over (**a**) 800-Cux-Zn-Al and (**b**) 800-Ce/Cux-Zn-Al mixed metal oxides; experimental conditions: mass of catalysts = 200 mg, [NH_3_] = 0.035 mol.%, [O_2_] = 20 mol.%, [N_2_] = balance, total flow rate = 100 cm^3^ min^−1^, WHSV = 500 mL min^−1^ g^−1^, temperature 350 °C.

The most desired, neutral, products of ammonia oxidation are dinitrogen and water vapor. However, due to the low dinitrogen selectivity observed in 800-Cux-Zn-Al and 800-Ce/Cux-Zn-Al, the generation of other nitrogen-based species, like NO, NO_2,_ or N_2_O, is possible. The catalysts’ efficiency, selectivity to dinitrogen, and side products at 350 °C are shown in Figure 6. As it was expected, apart from nitrogen, nitric oxide, nitrogen dioxide and nitrous oxide are detected. For the unmodified sample 800-Cux-Zn-Al, the selectivity to NO increases with the increase in Cu mol.% loading; however, no obvious trend is observed. For Ce-modified samples, the NO level slightly decreases with the increase in copper mol.% loading. Simultaneously, for the modified samples, the NO_2_ level increases, while the N_2_O level is lower than 5%. Both the catalysts’ selectivity and activity are more predictable in the case of modified samples than unmodified ones.

**Figure 7:**
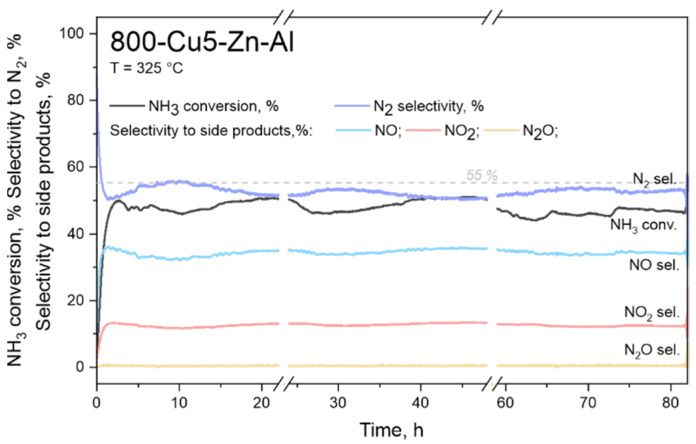
Results of the long-term stability tests for NH_3_-SCO performed on sample 800-Cu5-Zn-Al; experimental conditions: catalyst mass = 200 mg, [NH_3_] = 0.35 mol.%, [O_2_] = 20 mol.%, [N_2_] = 79.65 mol.%, total flow rate = 100 cm^3^ min^−1^; WHSV = 500 mL min^−1^ g^−1^, temperature 325 °C.

The results of the long-term stability test performed over 800-Cu5-Zn-Al at a temperature 325 °C are presented in Figure 7. Sample 800-Cu5-Zn-Al was chosen for this study due to the highest N2 selectivity in comparison to the side products. The ammonia conversion is approximately 50%, while selectivity to N_2_ is approximately 52–55%. Observed conversion and selectivity levels slightly vary in the range ± 5%. Similar trends were observed for the selectivity to side products that are: 35% (NO), 13% (NO_2_) and 1% (N_2_O). The slight changes of conversion/selectivity level could be related to detector limitations (FTIR). However, despite these slight changes of conversion/selectivity level, it could be seen that 800-Cu5-Zn-Al sample represents high thermal stability.

## 4. Discussion

Calcination of Cux-Zn-Al hydrotalcite-like materials with different copper mol.% of total cation content allows obtaining mixed metal oxides (800-Cux-Zn-Al and 800-Ce/Cux-Zn-Al) characterized by specific phase composition and physicochemical properties. The appearance of simple and complex oxide forms was expected due to the high calcination temperature. Basic material characterization with the use of XRD confirms the formation of CuO, ZnO, (Cu,Zn)Al_2_O_4_ spinel-like structures and, additionally, CeO_2_ oxides for cerium-modified samples. On the one hand, XRD measurements show high similarity between the samples, regardless of the chemical composition, while the additional measurements of H_2_-TPR show the correlation between Cu mol.% loading and formation of different CuO oxide forms, as well as sample reducibility. 

**Figure 8:**
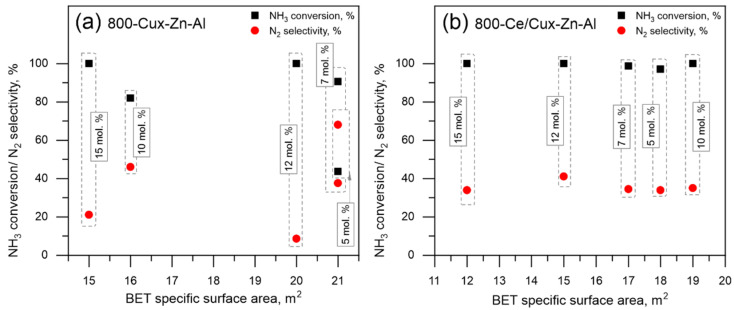
The catalytic efficiency of (**a**) 800-Cux-Zn-Al and (**b**) 800-Ce/Cux-Zn-Al at 350 °C vs. BET specific surface area.

Simultaneously, the characterization result and the comparison of catalytic activity increases with Cu mol.% loading, while catalyst selectivity to dinitrogen drops. Since the comparison of catalyst efficiency and specific surface area shows no visible correlation (Figure 8), the observed catalytic behavior should be connected to other aspects, such as the occurrence of CuO dispersed forms and samples modification by Ce. UV-Vis-DRS measurements confirm that samples with higher Cu mol.% loading—12 and 15 mol.% of total cation content are characterized by well-developed bulk-like CuO forms. At the same time, the catalytic activity of these samples is the highest, compared to other materials; while dinitrogen selectivity is the lowest and does not exceed 15 and 35% (at 350 °C) for 800-Cu12-Zn-Al and 800-Cu15-Zn-Al, respectively. Therefore, it could be reasoned that the occurrence of dispersed CuO phases favors process selectivity to dinitrogen, while bulk-like CuO phases favor sample activity.

Since the wet impregnation procedure includes sample recrystallization, the catalytic efficiency of 800-Ce/Cux-Zn-Al was expected to change in comparison to 800-Cux-Zn-Al. Indeed, all the Ce-modified samples reach 95–100% of NH_3_ conversion at 350 °C, which is within expectations. After material modification, process selectivity to dinitrogen was expected to decrease. However, the obtained results show a stable N_2_-level at 350 °C, approximately 35–40%, regardless of Cu mol.% loading. The observed dinitrogen selectivity at 350 °C is higher in comparison to un-modified materials that reach 100% ammonia conversion. The catalytic efficiency of 800-Ce/Cux-Zn-Al materials could be related to the activating effect of Cu-Ce-based oxide systems. Copper and cerium are known to form Cu-Ce redox couples (Cu^2+^-O-Ce^4+^) that, during the oxidation processes, are responsible for the activation of lattice oxygen. A similar synergistic effect was previously reported by Wang et al. [49], Lou et al. [50] and Gang et al. [31].

The analysis of catalytic results obtained at 350 °C showed that the main side products generated during oxidation are NO and N_2_O, wherein the selectivity to N_2_O seems to increase with the increase in Cu mol.% loading. Since the most probable mechanism of ammonia oxidation is internal selective ammonia oxidation (i-SCR), the occurrence of NO and NO_2_ as products is possible. According to the i-SCR mechanism, part of NH_3_ is oxidized to NO, and then NO is reduced by the remaining NH_3_ to dinitrogen and water vapor [49,51,52]_._ Thus, the catalytic efficiency of NH_3_-SCO catalysts will be limited by both NH_3_ oxidation and NO reduction, especially at low temperatures (up to 400 °C). The N_2_O is a possible side product of NO reduction. Increasing levels of N_2_O observed for both sets of samples suggest that, with increasing Cu mol.% loading, the reduction of NO by NH_3_ becomes more selective to nitrous oxide than N_2_. Therefore, the main goal of developing efficient NH_3_-SCO catalysts is to balance the reaction rate of NH_3_ oxidation and NO reduction while simultaneously increasing second process selectivity to N_2_. Our previous research [8] shows that iron-based oxide systems could improve the overall reaction selectivity. The results obtained at 350 °C, show the possibility of obtaining significantly higher selectivity to dinitrogen and significantly lower selectivity to nitrous oxide in comparison to both 800-Cux-Zn-Al and 800-Ce/Cux-Zn-Al material sets. At the same time, the iron-based oxides do not reach 100% of ammonia conversion.

Phase composition comparison between Cu-Zn-Al-Ox/(Ce) and Cu-Mg-Fe-Ox/(Ce) materials shows that calcination of hydrotalcite-like precursors leads to the formation of simple and complex oxide structures. Both dispersed and bulk-like CuO forms, responsible for catalysts activity and selectivity, were observed in both material sets. Therefore, the difference in catalytic efficiency between both material series is probably related to the nature of the copper oxides.

One of the main aspects differing the observed Cu-based oxides is reducibility (Table 5). It could be seen that in the case of Zn-Al-containing samples, the reduction of Cu^2+^ to Cu^0^ occurs at a lower temperature range in comparison to Mg-Fe-containing samples. The temperature shift between dispersed and bulk-like CuO phases is about 15–20 °C and 50–70 °C, respectively. Lowering the reduction temperature results in an increase in sample reducibility [53]. Comparing the reduction range of Cu^2+^ to Cu^0^ in spinel structures shows a significant difference between sample sets. The (Cu,Mg)Fe_2_O_4_-like structures are characterized by a considerably higher reduction range, approximately 580 °C, in comparison to the (Cu,Zn)Al_2_O_4_, with reduction occurring at approximately 230 °C. The hydrogen consumption measured up to 300 °C for Cux-Zn-Al-ox and Cux-Mg-Fe-ox materials is similar (except samples with the highest Cu mol.% loading) [8]. The results lead to the assumption that the amount of CuO forms, dispersed and bulk-like, observed for both series is equal. Therefore, the difference in catalytic efficiency could be connected to the specific behavior of these species, especially reducibility. It seems that the occurrence of easily reducible CuO species improves the catalysts’ activity. However, the relatively high process selectivity observed for the iron-magnesium mixed metal oxides seems to be related to the formation of spinel-like phases reducible at higher temperature ranges. The spinel oxide forms (Cu,Mg)Zn_2_O_4_ and (Cu,Mg)Fe_2_O_4_ are characterized by the same crystallographic structure, AB_2_O_4_; however, it is not possible to distinguish them from one another by simple characteristic methods, like XRD. However, H_2_-TPR measurements reveal that the iron-containing spinel forms are harder to reduce than zinc-based complex oxides. It suggests that the higher process selectivity observed for Cux-Mg-Fe-ox materials is related to the formation of hard to reduce spinel-like phases. Similar observations were previously reported by Basąg et al. [6], Gang et al. [31] or Liang et al. [19].

Since the BET-specific surface area of both material sets is similar, the reducibility of simple and complex oxide phases was recognized as the most important factor influencing catalyst efficiency in NH_3_-SCO. Thus, it is possible that the modification of copper-zinc–alumina oxides by iron implementation could improve catalytic efficiency of such mixed metal oxides at low-temperature ranges (up to 350 °C).

## 5. Conclusions

The presented work aimed to study the catalytic efficiency of 800-Cux-Zn-Al and 800-Ce/Cux-Zn-Al mixed metal oxides as catalysts for low temperature selective catalytic oxidation of ammonia to dinitrogen. The calcination of precursors (hydrotalcite-like materials), results in the formation of complex and simple oxide systems, including (Cu,Zn)Al_2_O_4_, CuO, ZnO and CeO_2_. Characterization result and catalytic test comparison allows to connect the occurrence of CuO (bulk-like and dispersed forms) and Cu-containing spinel forms with catalyst activity and selectivity, respectively. All tested materials exhibited high ammonia oxidation efficiency up to 350 °C; however, samples with higher Cu mol.% loading were more active while samples with lower Cu mol.% loading were more selective to dinitrogen. Sample modification by Ce improved catalytic efficiency.

## Figures and Tables

**Table 1:** Codes of calcined mixed metal oxides and their intended chemical composition expressed as mol.% of total metal cation composition, where the sum of Cu mol.%, Zn mol.%, Al mol.% and, if required, Ce mol.% is 100%.

Series 800-Cux-Zn-Al ‘Un-Modified’				Series 800-Ce/Cux-Zn-Al ‘Modified by Ce’				
Sample Code	Intended Chemical Composition, mol.%			Sample Code	Intended Chemical Composition, mol.%			
	Cu	Zn	Al		Cu	Zn	Al	Ce
800-Cu5-Zn-Al	5	62	33	800-Ce/Cu5-Zn-Al	4.8	59.2	31	5
800-Cu7-Zn-Al	7	60	33	800-Ce/Cu7-Zn-Al	6.8	57.2	31	5
800-Cu10-Zn-Al	10	57	33	800-Ce/Cu10-Zn-Al	9.6	54.4	31	5
800-Cu12-Zn-Al	12	55	33	800-Ce/Cu12-Zn-Al	11.5	52.5	31	5
800-Cu15-Zn-Al	15	52	33	800-Ce/Cu15-Zn-Al	14.4	49.6	31	5

**Table 2:** Chemical composition of catalysts, intended (int.) and calculated (cal.) Cu/Zn and Cu/Al molar ratio.

Sample	Molar Ratio Int.		EDS Chem. Com., wt. % ^1^			Molar Ratio Cal. ^2^		Ce wt. % ^1^ (EDS)
	Cu/Zn	Cu/Al	Cu	Zn	Al	Cu/Zn	Cu/Al	
800-Cu5-Zn-Al	0.08	0.15	5.5	68.9	25.6	0.08	0.09	-
800-Cu7-Zn-Al	0.12	0.21	7.9	69.7	22.3	0.12	0.15	-
800-Cu10-Zn-Al	0.17	0.30	13.7	62.5	23.8	0.22	0.24	-
800-Cu12-Zn-Al	0.22	0.36	13.0	65.1	21.9	0.20	0.25	-
800-Cu15-Zn-Al	0.29	0.45	17.1	62.4	20.4	0.28	0.36	-
800-Ce/Cu5-Zn-Al	0.08	0.15	5.4	64.1	22.1	0.09	0.10	8.43
800-Ce/Cu7-Zn-Al	0.12	0.21	7.1	60.5	24.9	0.12	0.12	7.61
800-Ce/Cu10-Zn-Al	0.17	0.31	12.3	55.2	23.1	0.23	0.23	9.39
800-Ce/Cu12-Zn-Al	0.22	0.37	12.2	62.4	18.0	0.20	0.29	7.41
800-Ce/Cu15-Zn-Al	0.29	0.46	18.0	58.7	15.2	0.31	0.50	8.14

^1^ wt. % of total metal cation content; ^2^ Calculated based on EDS measurements.

**Table 3:** Crystallite size, XRD intensity ratio of selected diffraction lines, direct bandgap energy and textural properties (text. prop.) of mixed metal oxides.

Sample	XRD Intensity Ratio ^1^			Crystallite Size, nm ^2^				*E*_g_^d 3^, eV	Text. Prop., m^2^ g^−1^	
	*I*_T_/*I*_Z_	*I*_G_/*I*_Z_	*I*_C_/*I*_Z_	CuO	ZnAl_2_O_4_	ZnO	CeO_2_		BET	t-Plot
800-Cu5-Zn-Al	0.07	0.19	-	26	19	44	-	3.24	21	6
800-Cu7-Zn-Al	0.11	0.22	-	38	15	32	-	3.23	21	6
800-Cu10-Zn-Al	0.22	0.27	-	41	20	42	-	3.20	16	2
800-Cu12-Zn-Al	0.19	0.29	-	31	23	35	-	3.21	20	5
800-Cu15-Zn-Al	0.29	0.36	-	38	18	40	-	3.21	15	2
800-Ce/Cu5-Zn-Al	0.06	0.19	0.11	30	22	38	23	3.21	18	4
800-Ce/Cu7-Zn-Al	0.09	0.21	0.12	31	22	49	28	3.17	17	5
800-Ce/Cu10-Zn-Al	0.20	0.27	0.17	38	23	49	29	3.21	19	3
800-Ce/Cu12-Zn-Al	0.17	0.27	0.10	41	23	48	26	3.23	15	4
800-Ce/Cu15-Zn-Al	0.26	0.34	0.10	36	27	49	19	3.17	12	3

^1^ Intensity ratio of characteristic XRD diffraction lines, *I*_T_—CuO, position 41° 2θ (*d* = 2.523, intensity 100%); *I*_G_—ZnAl_2_O_4_, position 43° 2θ (*d* = 2.438, intensity 100%); *I*_C_—CeO_2_, position 33° 2θ (*d* = 3.124, intensity 100%); *I*_Z_—ZnO, position 42° 2 Theta (*d* = 2.476, intensity 100%); ^2^ Crystallite size calculated based on characteristic XRD diffraction lines: CuO, position 41° 2θ (*d* = 2.523, intensity 100%); ZnAl_2_O_4_, position 36 and 43° 2 θ (*d* = 2.861, intensity 84% and *d* = 2.438, intensity 100%); ZnO, position 37, 40 and 42° 2 Theta (*d* = 2.814, intensity 57%, *d* = 2.603, intensity 44% and *d* = 2.476, intensity 100%); CeO_2_, position 33° 2θ (*d* = 3.124, intensity 100%); ^3^ Direct band gap energy estimated with use of Tauc’s plot.

**Table 4:** Theoretical and measured consumption of H_2_ during TPR calculated for Cu according to the elements amount determined by the EDS method.

Sample	Theoretical H_2_ Consumption ^1^, mmol g^−1^	Measured H_2_ Consumption (40–300 °C), mmol g^−1^	% of Theoretical H_2_ Consumption ^2^
800-Cu5-Zn-Al	0.864	0.402	47
800-Cu7-Zn-Al	1.258	0.528	42
800-Cu10-Zn-Al	2.161	0.888	41
800-Cu12-Zn-Al	2.046	0.846	41
800-Cu15-Zn-Al	2.703	1.269	47
800-Ce/Cu5-Zn-Al	0.845	0.281	33
800-Ce/Cu7-Zn-Al	1.114	0.442	40
800-Ce/Cu10-Zn-Al	1.938	0.834	43
800-Ce/Cu12-Zn-Al	1.928	0.832	43
800-Ce/Cu15-Zn-Al	2.830	1.125	40

^1^ Assuming that only Cu^2+^ forms occur and Cu is reduced completely to metallic form (Cu^2+^→Cu^0^); ^2^ % of theoretical H_2_ consumption according to the H_2_ consumption at 40–300 °C.

**Table 5:** Basic properties of 800-Cux-Zn-Al, 800-Ce/Cux-Zn-Al, 800-Cux-Mg-Fe, 800-Ce/Cux-Mg-Fe mixed metal oxide obtained by calcination of hydrotalcite-like compounds at 800 °C in air.

Samples Set	Phase Composition	Reduction of Cu^2+^, °C	BET Surface Area m^2^ g^−1^
800-Cux-Zn-Al	CuO, (Cu,Zn)Al_2_O_4_, ZnO	160–170 (D) 190–205 (B) 235 (S)	15–21
800-Ce/Cux-Zn-Al	CuO, (Cu,Zn)Al_2_O_4_, ZnO, CeO_2_	165–175 (D) 185–205 (B) 225–230 (S)	12–18
800-Cux-Mg-Fe	CuO, (Cu,Mg)Fe_2_O_4_, MgO, CeO_2_	180 (D) 250 (B) 580 (S)	6–18
800-Ce/Cux-Mg-Al	CuO, (Cu,Mg)Fe_2_O_4_, MgO, CeO_2_	195 (D) 260 (B) 580 (S)	6–18

(D) Dispersed CuO phase; (B) bulk-like CuO phase; (S) Cu^2+^ in spinel-like structure.

## Data Availability

Data is contained within the article or Supplementary Material.

## Supplementary Materials

The following are available online at https://www.mdpi.com/article/10.3390/ma14216581/s1, Table S1: Chemical composition of hydrotalcite-like compounds, intended (int.) and calculated Cu/Zn and Cu/Al molar ratio, Figure S1: Phase composition of HT-Cux-Zn-Al hydrotalcite-like compounds, Table S2: Chemical composition of precipitate (EDS, at. %) and M2+:M3+ molar ratio, Figure S2: Phase composition of precipitate, Figure S3: Textural properties of modified and not-modified samples; Ads—adsorption curve, Des—desorption curve, Figure S4: Micrographs of (a) 800-Cu5-Zn-Al, (b) 800-Cu7-Zn-Al, (c) 800-Cu10-Zn-Al, (d) 800-Cu12-Zn-Al, (e) 800-Ce/Cu5-Zn-Al, (f) 800-Ce/Cu7-Zn-Al, (g) 800-Ce/Cu10-Zn-Al, (h) 800-Ce/Cu12-Zn-Al, Figure S5: Micrographs of (a, b, c) 800-Cu10-Zn-Al and (d, e, f) 800-Ce/Cu10-Zn-Al; combined SE+BSE mode; Figure S6: UV-Vis-DRS spectra of (a) 800-Cux-Zn-Al and (b) 800-Ce/Cux-Zn-Al.

## References

[1] Dammers E., McLinden C.A., Griffin D., Shephard M.W., Van Der Graaf S., Lutsch E., Schaap M., Gainairu-Matz Y., Fioletov V., Van Damme M. (2019). NH_3_ emmisions from large point sources from CrIS and IASI satellite observations. Atmos. Chem. Phys. Discuss..

[2] Gómez-García M.A., Pitchon V., Kiennemann A. (2005). Pollution by nitrogen oxides: An approach to NOx abatement by using sorbing catalytic materials. Environ. Int..

[3] Huang R., Wu H., Yang L. (2020). Study on the ammonia emission characteristics in an ammonia-based WFGD system. Chem. Eng. J..

[4] Insausti M., Timmis R., Kinnersley R., Rufino M.C. (2020). Advances in sensing ammonia from agricultural sources. Sci. Total Environ..

[5] Jabłońska M., Palkovits R. (2016). Copper based catalysts for the selective ammonia oxidation into nitrogen and water vapour-Recent trends and open challenges. Appl. Catal. B Environ..

[6] Basąg S., Piwowarska Z., Kowalczyk A., Węgrzyn A., Baran R., Gil B., Michalik M., Chmielarz L. (2016). Cu-Mg-Al hydrotalcite-like materials as precursors of effective catalysts for selective oxidation of ammonia to dinitrogen—The influence of Mg/Al ratio and calcination temperature. Appl. Clay Sci..

[7] Gao F., Liu Y., Sani Z., Tang X., Yi H., Zhao S., Yu Q., Zhou Y. (2021). Advances in selective catalytic oxidation of ammonia (NH_3_-SCO) to dinitrogen in excess oxygen: A review on typical catalysts, catalytic performances and reaction mechanisms. J. Environ. Chem. Eng..

[8] Górecka S., Pacultová K., Górecki K., Smýkalová A., Pamin K., Obalová L. (2020). Cu-Mg-Fe-O-(Ce) complex oxides as catalysts of selective catalytic oxidation of ammonia to dinitrogen (NH_3_-SCO). Catalysts.

[9] Chmielarz L., Jabłońska M. (2015). Advances in selective catalytic oxidation of ammonia to dinitrogen: A review. RSC Adv..

[10] Jabłońska M. (2015). Selective catalytic oxidation of ammonia into nitrogen and water vapour over transition metals modified Al_2_O_3_, TiO_2_ and ZrO_2_. Chem. Pap..

[11] Jabłońska M., Nocuń M., Gołąbek K., Palkovits R. (2017). Effect of preparation procedures on catalytic activity and selectivity of copper-based mixed oxides in selective catalytic oxidation of ammonia into nitrogen and water vapour. Appl. Surf. Sci..

[12] Wang H., Zhang Q., Zhang T., Wang J., Wei G., Liu M., Ning P. (2019). Structural tuning and NH_3_-SCO performance optimization of CuO-Fe_2_O_3_ catalysts by impact of thermal treatment. Appl. Surf. Sci..

[13] Zhao H., Qu Z., Sun H. (2020). Rational design of spinel CoMn_2_O_4_ with Co-enriched surface as high-activity catalysts for NH_3_-SCO reaction. Appl. Surf. Sci..

[14] Pérez-Ramírez J., Kondratenko E.V. (2007). Mechanism of ammonia oxidation over oxides studied by temporal analysis of products. J. Catal..

[15] Jabłońska M. (2015). TPR study and catalytic performance of noble metals modified Al_2_O_3_, TiO_2_ and ZrO_2_ for low-temperature NH_3_-SCO. Catal. Commun..

[16] Wang F., Zhu Y., Li Z., Shan Y., Shan W., Shi X., Yu Y., Zhang C., Li K., Ning P. (2020). Promoting effect of acid sites on NH_3_-SCO activity with water vapor participation for Pt-Fe/ZSM-5 catalyst. Catal. Today.

[17] Lin M., An B., Takei T., Shishido T., Ishida T., Haruta M., Murayama T. (2020). Features of Nb_2_O_5_ as a metal oxide support of Pt and Pd catalysts for selective catalytic oxidation of NH_3_ with high N_2_ selectivity. J. Catal..

[18] Shin J.H., Kim G.J., Hong S.C. (2020). Reaction properties of ruthenium over Ru/TiO_2_ for selective catalytic oxidation of ammonia to nitrogen. Appl. Surf. Sci..

[19] Liang C., Li X., Qu Z., Tade M., Liu S. (2012). The role of copper species on Cu/γ-Al_2_O_3_ catalysts for NH_3_-SCO reaction. Appl. Surf. Sci..

[20] Chang S., Harle G., Ma J., Yi J. (2020). The effect of textural properties of CeO_2_-SiO_2_ mixed oxides on NH_3_-SCO activity of Pt/CeO_2_-SiO_2_ catalyst. Appl. Catal. A Gen..

[21] Lee S.M., Lee H.H., Hong S.C. (2014). Influence of calcination temperature on Ce/TiO_2_ catalysis of selective catalytic oxidation of NH_3_ to N_2_. Appl. Catal. A Gen..

[22] Guo J., Yang W., Zhang Y., Gan L., Fan C., Chen J., Peng Y., Li J. (2020). A multiple-active-site Cu/SSZ-13 for NH_3_-SCO: Influence of Si/Al ratio on the catalytic performance. Catal. Commun..

[23] Rutkowska M., Pacia I., Basąg S., Kowalczyk A., Piwowarska Z., Duda M., Tarach K.A., Góra-Marek K., Michalik M., Díaz U. (2017). Catalytic performance of commercial Cu-ZSM-5 zeolite modified by desilication in NH_3_-SCR and NH_3_-SCO processes. Microporous Mesoporous Mater..

[24] Guo J., Peng Y., Zhang Y., Yang W., Gan L., Li K., Chen J., Li J. (2019). Comparison of NH_3_-SCO performance over CuOx/H-SSZ-13 and CuOx/H-SAPO-34 catalysts. Appl. Catal. A Gen..

[25] Kowalczyk A., Święs A., Gil B., Rutkowska M., Piwowarska Z., Borcuch A., Michalik M., Chmielarz L. (2018). Effective catalysts for the low-temperature NH_3_-SCR process based on MCM-41 modified with copper by template ion-exchange (TIE) method. Appl. Catal. B Environ..

[26] Chen C., Cao Y., Liu S., Chen J., Jia W. (2019). The catalytic properties of Cu modified attapulgite in NH_3_–SCO and NH_3_–SCR reactions. Appl. Surf. Sci..

[27] Curtin T., Lenihan S. (2003). Copper exchanged beta zeolites for the catalytic oxidation of ammonia. Chem. Commun..

[28] Curtin T., O’Regan F., Deconinck C., Knüttle N., Hodnett B.K. (2000). The catalytic oxidation of ammonia: Influence of water and sulfur on selectivity to nitrogen over promoted copper oxide/alumina catalysts. Catal. Today.

[29] Górecka S., Pacultová K., Smýkalová A., Fridrichová D., Górecki K., Rokicińska A., Kuśtrowski P., Žebrák R., Obalová L. (2021). Role of the Cu content and Ce activating effect on catalytic performance of Cu-Mg-Al and Ce/Cu-Mg-Al oxides in ammonia selective catalytic oxidation. Appl. Surf. Sci..

[30] Gang L., Anderson B.G., Van Grondelle J., Van Santen R.A. (2000). NH_3_ oxidation to nitrogen and water at low temperatures using supported transition metal catalysts. Catal. Today.

[31] Gang L., Van Grondelle J., Anderson B.G., Van Santen R.A. (1999). Selective low temperature NH_3_ oxidation to N_2_ on copper-based catalysts. J. Catal..

[32] Lippits M.J., Gluhoi A.C., Nieuwenhuys B.E. (2008). A comparative study of the selective oxidation of NH_3_ to N_2_ over gold, silver and copper catalysts and the effect of addition of Li_2_O and CeOx. Catal. Today.

[33] He S., Zhang C., Yang M., Zhang Y., Xu W., Cao N., He H. (2007). Selective catalytic oxidation of ammonia from MAP decomposition. Sep. Purif. Technol..

[34] Mayer R.W., Hävecker M., Knop-Gericke A., Schlügl R. (2001). Investigation of ammonia oxidation over copper with in situ NEXAFS in the soft X-ray range: Influence of pressure on the catalyst performance. Catal. Lett..

[35] Chmielarz L., Jabłońska M., Strumiński A., Piwowarska Z., Wegrzyn A., Witkowski S., Michalik M. (2013). Selective catalytic oxidation of ammonia to nitrogen over Mg-Al, Cu-Mg-Al and Fe-Mg-Al mixed metal oxides doped with noble metals. Appl. Catal. B Environ..

[36] Trombetta M., Ramis G., Busca G., Montanari B., Vaccari A. (1997). Ammonia adsorption and oxidation on Cu/Mg/Al mixed oxide catalysts prepared via hydrotalcite-type precursors. Langmuir.

[37] Jabłońska M., Palomares A.E., Chmielarz L. (2013). NOx storage/reduction catalysts based on Mg/Zn/Al/Fe hydrotalcite-like materials. Chem. Eng. J..

[38] Zhang Y.S., Li C., Yu C., Tran T., Guo F., Yang Y., Yu J., Xu G. (2017). Synthesis, characterization and activity evaluation of Cu-based catalysts derived from layered double hydroxides (LDHs) for DeNOx reaction. Chem. Eng. J..

[39] Wang C., Yang S., Chang H., Peng Y., Li J. (2013). Structural effects of iron spinel oxides doped with Mn, Co, Ni and Zn on selective catalytic reduction of NO with NH_3_. J. Mol. Catal. A Chem..

[40] Marchi A.J., Di Cosimo J.I., Apesteguia C.R. (1993). Influence of the chemical composition on the preparation of Cu-Co-Zn-Al mixed oxide catalysts with high metal dispersion. New Front. Catal..

[41] Obalová L., Karásková K., Wach A., Kustrowski P., Mamulová-Kutláková K., Michalik S., Jirátová K. (2013). Alkali metals as promoters in Co-Mn-Al mixed oxide for N_2_O decomposition. Appl. Catal. A Gen..

[42] Basąg S., Kovanda F., Piwowarska Z., Kowalczyk A., Pamin K., Chmielarz L. (2017). Hydrotalcite-derived Co-containing mixed metal oxide catalysts for methanol incineration: Role of cobalt content, Mg/Al ratio and calcination temperature. J. Therm. Anal. Calorim..

[43] Shannon R.D. (1976). Revised effective ionic Radii and systematic studies of interatomic distances in halides and chalcogenides. Acta Crystallogr..

[44] Da Silva W.J., Da Silva M.R., Takashima K. (2015). Preparation and characterization of Zno/CuO semiconductor and photocatalytic activity on the decolorization of direct red 80 azodye. J. Chil. Chem. Soc..

[45] Chen K., Zhang T., Chen X., He Y., Liang X. (2018). Model construction of micro-pores in shale: A case study of Silurian Longmaxi Formation shale in Dianqianbei area, SW China. Pet. Explor. Dev..

[46] Basąg S., Kocoł K., Piwowarska Z., Rutkowska M., Baran R., Chmielarz L. (2017). Activating effect of cerium in hydrotalcite derived Cu–Mg–Al catalysts for selective ammonia oxidation and the selective reduction of NO with ammonia. React. Kinet. Mech. Catal..

[47] Jabłońska M., Chmielarz L., Węgrzyn A., Guzik K., Piwowarska Z., Witkowski S., Walton R.I., Dunne P.W., Kovanda F. (2013). Thermal transformations of Cu-Mg (Zn)-Al(Fe) hydrotalcite-like materials into metal oxide systems and their catalytic activity in selective oxidation of ammonia to dinitrogen. J. Therm. Anal. Calorim..

[48] Lee S.M., Hong S.C. (2015). Promotional effect of vanadium on the selective catalytic oxidation of NH_3_ to N_2_ over Ce/V/TiO_2_ catalyst. Appl. Catal. B Environ..

[49] Wang Z., Qu Z., Quan X., Li Z., Wang H., Fan R. (2013). Selective catalytic oxidation of ammonia to nitrogen over CuO-CeO_2_ mixed oxides prepared by surfactant-templated method. Appl. Catal. B Environ..

[50] Lou J.C., Hung C.M., Yang S.F. (2004). Selective Catalytic Oxidation of Ammonia over Copper-Cerium Composite Catalyst. J. Air Waste Manag. Assoc..

[51] Zhang X., Wang H., Wang Z., Qu Z. (2018). Adsorption and surface reaction pathway of NH_3_ selective catalytic oxidation over different Cu-Ce-Zr catalysts. Appl. Surf. Sci..

[52] Zhang L., He H. (2009). Mechanism of selective catalytic oxidation of ammonia to nitrogen over Ag/Al_2_O_3_. J. Catal..

[53] Puigdollers A.R., Schlexer P., Tosoni S., Pacchioni G. (2017). Increasing oxide reducibility: The role of metal/oxide interfaces in the formation of oxygen vacancies. ACS Catal..

