# Supplementary material for: Catalytic Oxidation of Ammonia over Cerium-Modified Copper Aluminium Zinc Mixed Oxides
Source: Materials (Basel). 2021 Nov 3;14(21):6581. doi: 10.3390/ma14216581 (PMC8585330; doi:10.3390/ma14216581)
Supplement: Supplementary file 1 [file materials-14-06581-s001.zip › materials-1431162-supplementary.pdf]

materials

Catalytic Oxidation of Ammonia over Cerium-Modified Copper Aluminium Zinc Mixed Oxides

Sylwia Górecka [1,*], Kateřina Pacultová [1], Dagmar Fridrichová [1], Kamil Górecki [1], Tereza Bílková [1], Radim Žebrák [2] and Lucie Obalová [1]

1 Institute of Environmental Technology, CEET, VSB-Technical University of Ostrava, 17. listopadu 15/2172, 708 00 Ostrava-jih, Czech Republic; katerina.pacultova@vsb.cz (K.P.); dagmar.fridrichova@vsb.cz (D.F.); kamil.maciej.gorecki@vsb.cz (K.G.); tereza.bilkova@vsb.cz (T.B.); lucie.obalova@vsb.cz (L.O.)
2 Dekonta a.s., Dřetovice 109, 273 42 Stehelčeves, Czech Republic, radim.zebrak@dekonta.cz
* Correspondence: sylwia.gorecka@vsb.cz; Tel.: +420-597-327-315

Table S1. Chemical composition of hydrotalcite-like compounds, intended (int.) and calculated Cu/Zn and Cu/Al molar ratio.

Sample code	Int. chem. com., mol. % [1]			Molar ratio int.		AAS chem. com., wt. %			Molar ratio [2] calculated	
	Cu	Zn	Al	Cu/Zn	Cu/Al	Cu	Zn	Al	Cu/Zn	Cu/Al
HT-Cu5-Zn-Al	5	62	33	0.08	0.15	3.10	38.8	7.8	0.08	0.17
HT-Cu7-Zn-Al	7	60	33	0.12	0.21	3.80	38.8	7.7	0.10	0.21
HT-Cu10-Zn-Al	10	57	33	0.17	0.30	7.90	32.1	8.2	0.25	0.41
HT-Cu12-Zn-Al	12	55	33	0.22	0.36	8.40	35.3	7.8	0.24	0.46
HT-Cu15-Zn-Al	15	52	33	0.29	0.45	10.50	32.8	7.6	0.33	0.59

[1] mol.% of total cation content; [2] calculated on the basis of AAS measurements results.

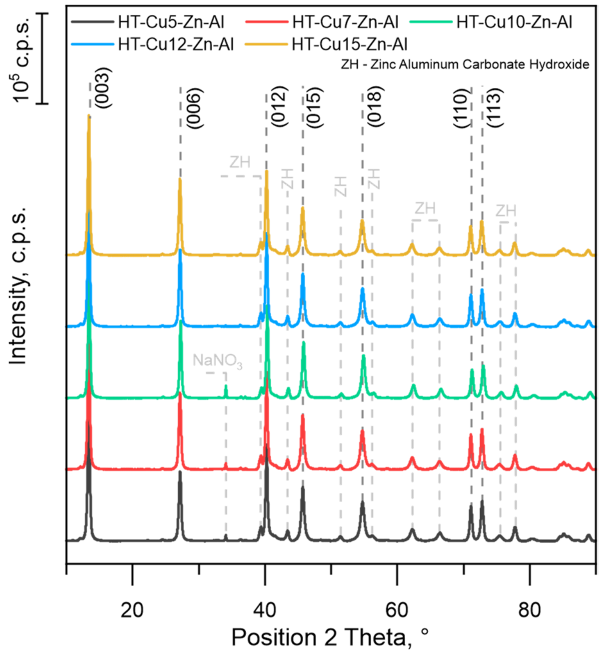

Figure S1. Phase composition of HT-Cux-Zn-Al hydrotalcite-like compounds.

Table S2. Chemical composition of precipitate (EDS, at. %) and M^{2+}:M^{3+} molar ratio.

Sample	EDS chemical composition, at. % [1]			M^{2+} : M^{3+} ratio
	Cu	Zn	Al	
800-Cu5-Zn-Al	2.9	29.5	67.6	1:2.1
800-Cu7-Zn-Al	3.0	27.5	69.4	1:2.3
800-Cu10-Zn-Al	3.6	31.9	64.5	1:1.8
800-Cu12-Zn-Al	2.9	30.5	66.6	1:2.0
800-Cu15-Zn-Al	2.5	27.1	70.4	1:2.4
800-Ce/Cu5-Zn-Al	2.8	30.9	66.3	1:2.0
800-Ce/Cu7-Zn-Al	3.6	31.7	64.7	1:1.8
800-Ce/Cu10-Zn-Al	4.7	30.4	64.9	1:1.8
800-Ce/Cu12-Zn-Al	3.4	31.6	65.0	1:1.8
800-Ce/Cu15-Zn-Al	3.6	28.4	68.0	1:2.1

[1] at. % of total metal amount.

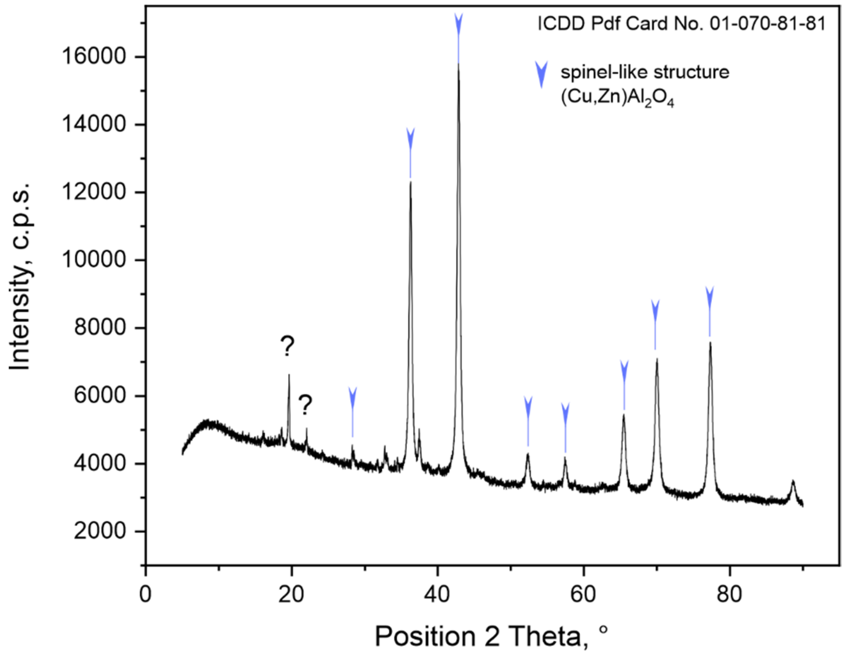

Figure S2. Phase composition of precipitate.

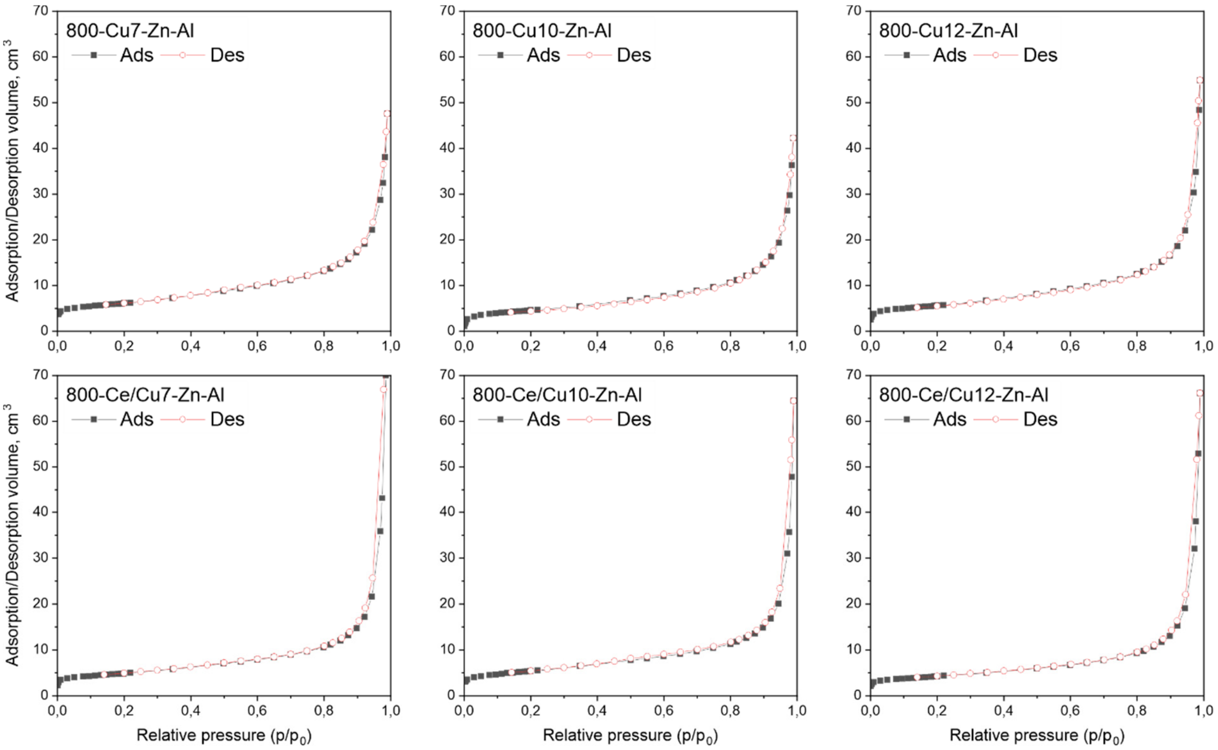

Figure S3. Textural properties of modified and not-modified samples; Ads – adsorption curve, Des – desorption curve.

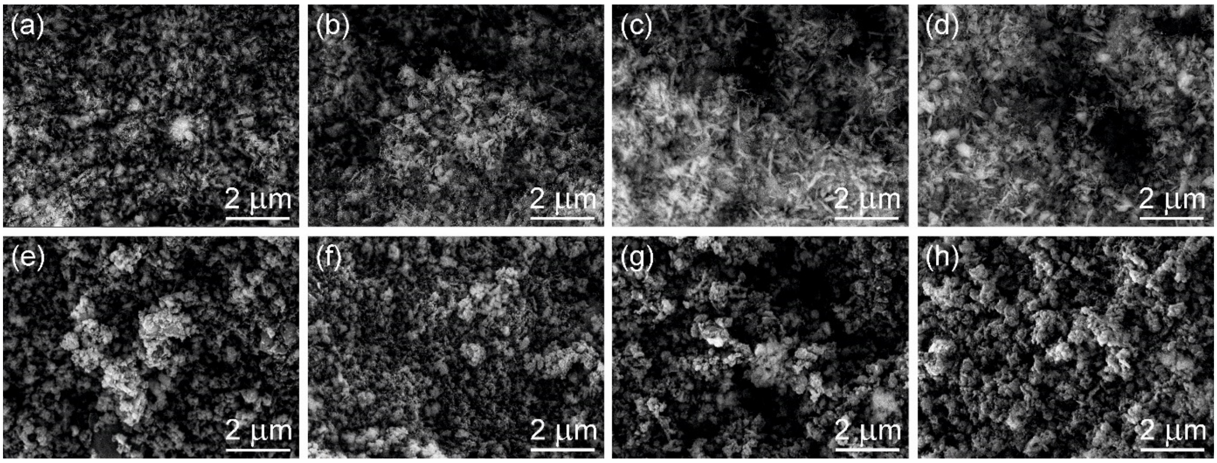

Figure S4. Micrographs of (**a**) 800-Cu5-Zn-Al, (**b**) 800-Cu7-Zn-Al, (**c**) 800-Cu10-Zn-Al, (**d**) 800-Cu12-Zn-Al, (**e**) 800-Ce/Cu5-Zn-Al, (**f**) 800-Ce/Cu7-Zn-Al, (**g**) 800-Ce/Cu10-Zn-Al, (**h**) 800-Ce/Cu12-Zn-Al.

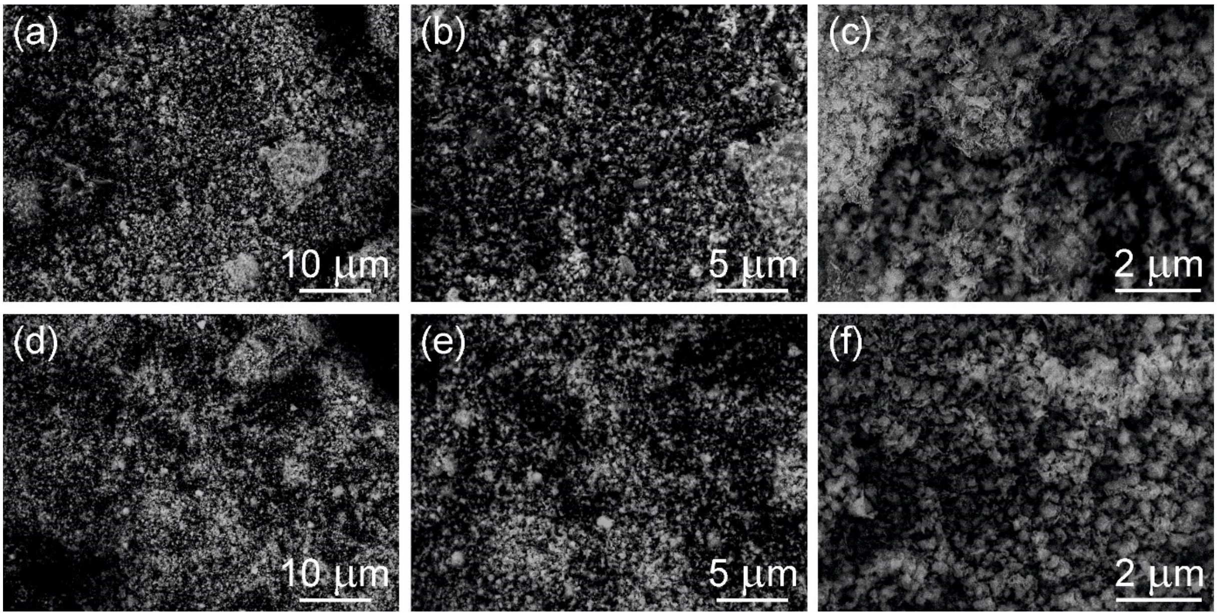

Figure S5. Micrographs of (**a**, **b**, **c**) 800-Cu10-Zn-Al and (**d**, **e**, **f**) 800-Ce/Cu10-Zn-Al; combined SE+BSE mode.

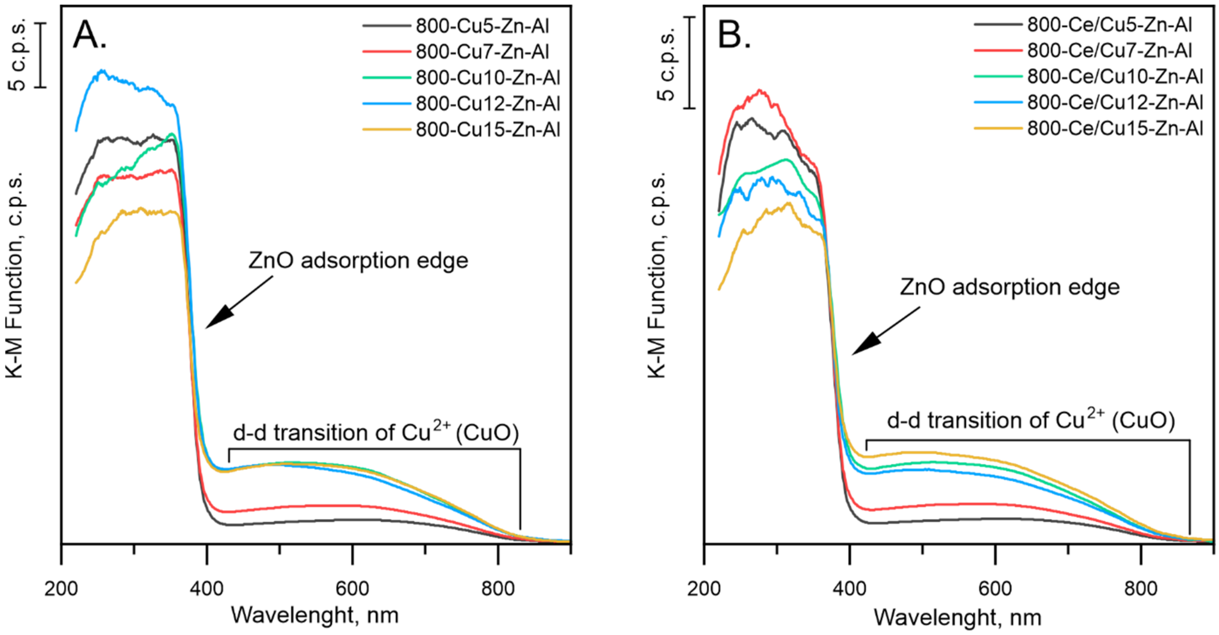

Figure S6. UV-Vis-DRS spectra of (**a**) 800-Cux-Zn-Al and (**b**) 800-Ce/Cux-Zn-Al.

© 2020 by the authors. Submitted for possible open access publication under the terms and conditions of the Creative Commons Attribution (CC BY) license (http://creativecommons.org/licenses/by/4.0/).
